# Increase of the Intracellular Zinc Concentration Leads to an Activation and Internalisation of the Epidermal Growth Factor Receptor in A549 Cells

**DOI:** 10.3390/ijms22010326

**Published:** 2020-12-30

**Authors:** Lisa-Marie Barth, Lothar Rink, Inga Wessels

**Affiliations:** Institute of Immunology, Medical Faculty, RWTH Aachen University, Pauwelsstraße 30, D-52074 Aachen, Germany; lisa.barth@rwth-aachen.de

**Keywords:** zinc, EGFR, endocytosis, signalling, A549 alveolar cells

## Abstract

(1) Background: Zinc is suggested to play a major role in epidermal growth factor (EGF)-induced cell regeneration and proliferation. To deepen the knowledge on the underlying mechanisms zinc’s effects on the epidermal growth factor receptor (EGFR) activation and its endocytosis was investigated in the alveolar carcinoma cell line A549. (2) Methods: An increase of intracellular zinc was generated by adding zinc extracellularly compared to the intracellular release of zinc from zinc-binding proteins by stimulation with a nitric oxide donor. Zinc-initiated EGFR phosphorylation was checked by Western blotting and receptor endocytosis assays were performed by using flow cytometry. (3) Results: Besides a dose-dependent EGFR phosphorylation, a dose- and time dependent significant receptor internalisation was initiated by both types of zinc increases. In addition, both increased intracellular zinc levels further promoted EGF-induced EGFR phosphorylation and internalisation. (4) Conclusion: This report confirms a transactivating effect of zinc on the EGFR for A549 cells and is the first describing an influence of zinc on the EGFR endocytosis. The transferability of the fine-tuning of EGFR-induced signalling by zinc needs to be verified in vivo, but the presented data underline that zinc might be helpful during treatment of disturbed regeneration and tissue repair.

## 1. Introduction

By supporting cell growth and wound healing processes, zinc is an essential trace element involved in the maintenance of cellular systems. Additionally, in the human airway epithelium a predominant cytoprotective role is attributed to zinc. Zinc exemplarily prevents apoptosis by protecting cells against oxidative stress and is further involved in proliferation and reepithelization by promoting the activation of growth factor receptors [1,2]. Previous studies paid particular attention to the epidermal growth factor receptor (EGFR/ErbB-1), which belongs to a family of receptor tyrosine kinases and is physiologically involved in the regulation of cell migration, proliferation, differentiation and apoptosis. Augmented EGFR signalling, due to receptor mutation, overexpression and transactivation, is though known to be associated with progression, invasion and metastasis of several cancer types [3]. One well known example is lung cancer, in which EGFR overexpression was associated with poor prognosis [4]. Moreover, EGFR overexpression and increased recycling by transforming growth factor-α (TGF-α) has been associated with pulmonary fibrosis [5]. In addition to its inactivation, the downregulation of the EGFR expression might thus be a therapeutic approach regarding those pulmonal diseases. Former studies indicate that zinc transactivates the EGFR; however, the mechanisms of receptor activation and the induced signalling effects by zinc vary strongly between different experimental settings and cell types [6,7,8,9,10,11]. Since the EGFR gained great importance in recent decades and the influence of zinc on its function has not yet been fully understood, examination continues to be reasonable and is of especial interest regarding pulmonal cells.

In the presented report the effects of adding zinc extracellularly compared to inducing its release from intracellular stores on the activation and internalisation of the EGFR in A549 cells were compared, as a novel approach. The A549 cell line is an alveolar carcinoma cell line and a well-established model for non-small cell lung cancer [12]. As A549 cells express a wild type EGFR, they probably represent a suitable model for investigating zinc’s role in physiological EGFR-induced signalling as well [12,13]. In contrast to former works, particularly focusing on the influence of zinc on downstream signalling pathway [6,7,8], the focus of our project is to address the impact of zinc on EGFR endocytosis. EGFR endocytosis, as a consequence of receptor activation or transactivation, is a pivotal mechanism modulating signal transduction, by introducing receptor degradation or recycling [3]. Thus, to broaden the understanding of the known mitogen potential resulting from a zinc-mediated transactivation of this receptor, it is necessary to direct the focus on the influence of zinc on the EGFR internalisation. In the following, our study provides first insights into this topic.

## 2. Results

### 2.1. Zinc and Pyrithione as Well as SNOC Increase the Intracellular Zinc Concentration

In addition to specific importers and exporters, the free intracellular zinc content is regulated by zinc binding proteins, from which a zinc release is mediated through the oxidation of zinc/thiolate-clusters [14]. In the conducted experiments, apart from adding zinc together with its ionophore extracellularly, an increase in free intracellular zinc was generated by SNOC. SNOC S-nitrosates the zinc/thiolate clusters of proteins, which causes a more physiological zinc increase from intracellular stores [15,16].

**Figure 1:**
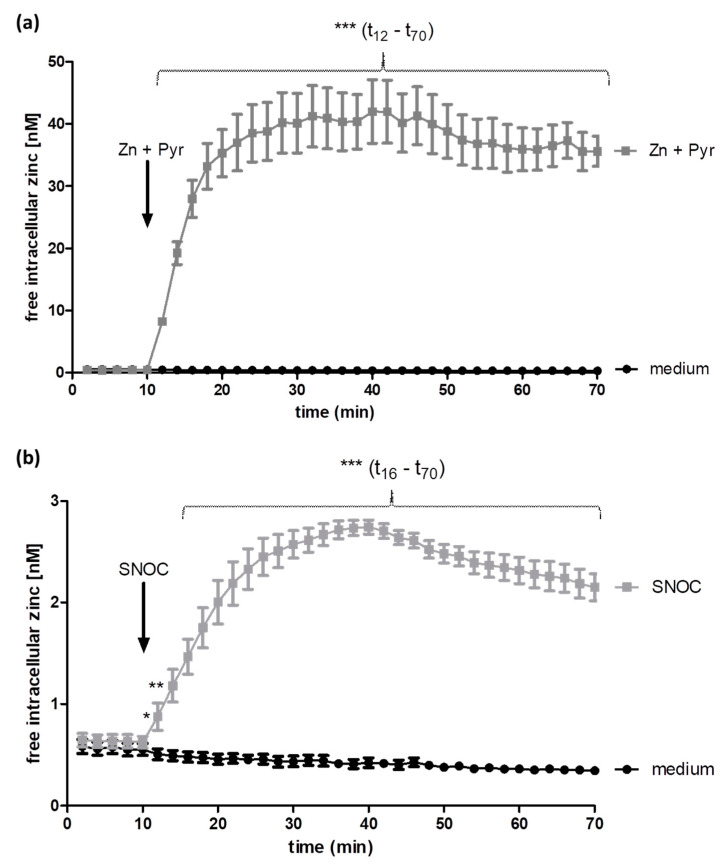
Increase of intracellular free zinc levels. Incubation with zinc (Zn, 50 µM) and pyrithione (Pyr, 0.5 µM) (**a**) or SNOC (2 mM) (**b**) induces an increase of the free intracellular zinc concentration. After assessing a base line, the kinetic measurement was started immediately after adding zinc and pyrithione or SNOC (arrow) and was conducted by using a well plate reader. Data of *n* = 6–7 independent experiments are shown as means +/− SEM. Statistical significances, calculated by students *t*-test, are depicted as * (*p* < 0.05), ** (*p* < 0.01) or *** (*p* < 0.001). Braces show significant elevated zinc levels (***) for all included time points.

To define the time course of the alterations in intracellular zinc levels mediated by zinc and pyrithione compared to SNOC, the free intracellular zinc concentration was recorded via FluoZin-3 AM for 60 min after adding the stimulants. Treating A549 cells with zinc and the ionophore pyrithione led immediately to a strong increase of the intracellular free zinc level compared to untreated cells (medium) (Figure 1a). With a steep increase, intracellular zinc content rose to over 40 nM, whereas untreated cells (medium) showed a low and constant free zinc level (below 1 nM). The free zinc concentration reached its maximum within 30 min and stayed largely stable on this high level until the analyses was stopped after 70 min. SNOC caused a rise of the intracellular zinc concentration to over 2.5 nM, while untreated cells (medium) maintained their initial free zinc level (below 1 nM) (Figure 1b). The increase in intracellular zinc level, elicited by SNOC, had a lower magnitude, compared to the stimulation with zinc and pyrithione, reached the highest zinc concentration after 30 min of stimulation and subsequently fell slightly until the end of the experiment. Concluding, zinc and pyrithione as well as SNOC cause a significant increase in intracellular free zinc in A549 cells.

### 2.2. Increase of the Free Intracellular Zinc Level Activates the EGFR

**Figure 2:**
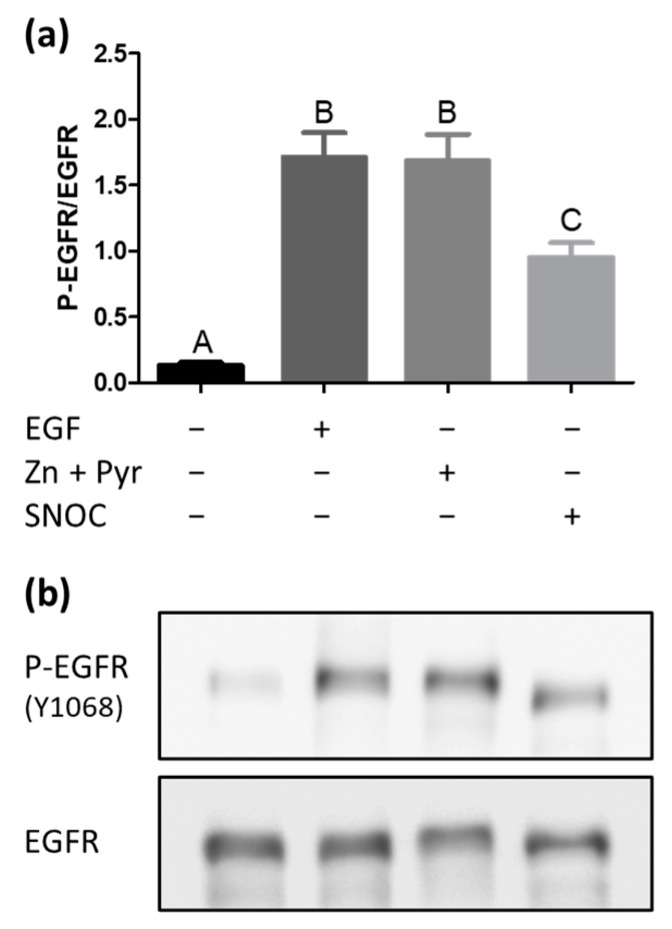
Zinc induces EGFR phosphorylation. The effect of EGF, zinc (Zn) and pyrithione (Pyr) or SNOC on the tyrosine 1068 phosphorylation of the EGFR is shown. A549 cells were stimulated for 60 min with EGF (25 ng/mL), zinc (50 µM) and pyrithione (0.5 µM) or SNOC (2 mM) and phosphorylation effects were quantified by Western blotting. Data of *n* = 12 independent experiments are shown as means + SEM (**a**). One representative Western blot is presented (**b**). Statistical significances were calculated with one-way Anova and Tukey’s post-hoc test (*p* < 0.05). Means not sharing any letter are significantly different (A–C).

To investigate whether the increases of the free intracellular zinc concentration resulted in activation of the EGFR, A549 cells were stimulated with either zinc and pyrithione, SNOC, or EGF for 60 min. Subsequently, the phosphorylation state of the EGFR at tyrosine 1068, which is one of the main autophosphorylation sites known for this receptor, was measured [6,7,8,9]. In unstimulated and starved cells, the EGFR was predominantly found in a low phosphorylation state (Figure 2). Stimulation with the natural ligand EGF led to a phosphorylation of the tyrosine 1068, which was approximately ten times higher compared to the receptor phosphorylation in unstimulated cells. Beyond that, the magnitude of phosphorylation induced by stimulation with zinc and pyrithione was comparable to the effect achieved by EGF itself. Adding SNOC to A549 cells also caused a lower but nevertheless significant phosphorylation of the tyrosine 1068. The total concentration of the EGFR was not affected by any of the stimulants (data not shown). In summary, an increase of the intracellular free zinc concentration by SNOC or zinc and pyrithione stimulation for 60 min leads to a significant activation of the EGFR.

### 2.3. Increase of the Intracellular Zinc Level Provokes EGFR Internalisation

**Figure 3:**
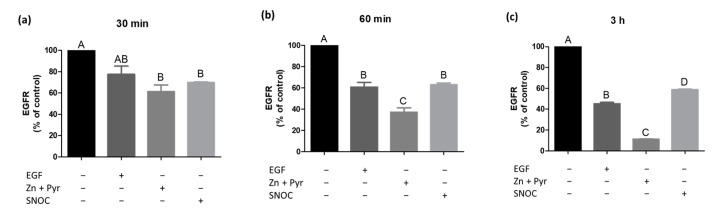
Zinc reduces EGFR surface expression. The effect of EGF (10 ng/mL), zinc (Zn, 50 µM) and pyrithione (Pyr, 0.5 µM) or SNOC (2 mM) on the surface expression of the EGFR is shown. A549 cells were stimulated for 30 min (**a**), 60 min (**b**) or 3 h (**c**) and measurements were performed by using flow cytometry. Data of *n* = 3 independent experiments are shown as means + SEM. Statistical significances were calculated with one-way Anova and Tukey’s post-hoc test (*p* < 0.05). Means not sharing any letter are significantly different (A–D).

Since our results revealed that zinc from extracellular as well as intracellular sources transactivates the EGFR, a subsequent receptor endocytosis was very likely. However, the zinc-induced EGFR endocytosis was to our knowledge not proven in previous work so far. Thus, time-dependent effects of zinc on EGFR levels on the cell surface were measured. A549 cells were stimulated continuously with EGF, zinc and pyrithione or SNOC. Figure 3a–c show a time- and stimulus-dependent decrease in EGFR surface expression. As the total cellular EGFR protein levels stayed constant for all stimulants in our experiments, the observed changes most probably depend on receptor internalisation processes rather than on receptor shedding. In the following, effects are thus described as results of receptor endocytosis.

While we had confirmed receptor activation after 60 min stimulation (Figure 2), internalisation of the receptor was already detectable after 30 min (Figure 3a). In particular, the incubation with zinc and pyrithione led to a strong receptor endocytosis of 40%. SNOC treatment for 30 min triggered an EGFR endocytosis of 30% and stimulation with EGF caused a receptor internalisation of 20%. The latter was, however, not yet significant (Figure 3a). For all stimulants, treatment for 60 min led to a greater and thus more significant internalisation effect compared to the 30 min incubation. Endocytosis induced by SNOC progressed slower (10% in 30 min) than in case of zinc and pyrithione or EGF, where 20% of the EGFR were already internalized within 30 min (Figure 3b). A constant EGFR internalisation was measured for all stimulants during the 3 h stimulation (Figure 3c). It can be noted that the internalisation was attenuated over time, especially regarding SNOC. After 3 h, approximately 40%, 55% and 90% of the EGFR was lost from the surface in case of SNOC, EGF or zinc and pyrithione treatment, respectively underlining the massive effect of changes in extracellular zinc levels on receptor endocytosis (Figure 3c).

### 2.4. EGF Stimulation Does Not Increase the Intracellular Zinc Concentration

**Figure 4:**
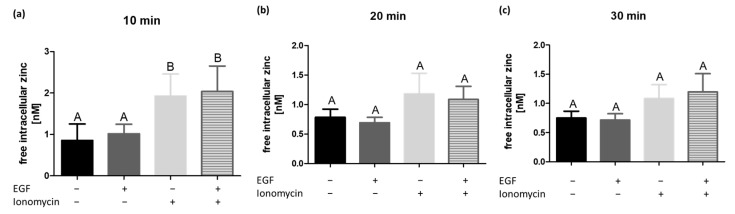
EGF does not induce changes of the intracellular free zinc level. The effect of a stimulation with EGF (25 ng/mL), ionomycin (0.5 µM) and a combination of both on the free intracellular zinc concentration is shown. A549 cells were stimulated for 10 min (**a**), 20 min (**b**) or 30 min (**c**) and measurements were performed by using flow cytometry. Data of *n* = 4 independent experiments are shown as means + SEM. Statistical significances were calculated using one-way Anova and Tukey’s post-hoc test. Means not sharing any letter are significantly different (A, B).

Since zinc is often involved in signalling processes [17] and the afore mentioned data revealed that effects of zinc and EGF are similar to each other (Figure 2 and Figure 3), we assumed EGF itself might induce an intracellular zinc increase. A physiological participation of a zinc wave in EGF-induced EGFR activation was thus tested in the following experiment (Figure 4). By treating A549 cells with EGF for different time points, no significant changes of the free intracellular zinc concentration could be quantified with FluoZin-3 AM. The calcium ionophore ionomycin is known to trigger a zinc release from the endoplasmic reticulum [18] and showed accordingly an increase of the free intracellular zinc concentration for all timepoints. A simultaneous treatment with EGF and ionomycin did not enhance the intracellular free zinc concentration obtained by ionomycin treatment alone. Concluding, the EGF-induced EGFR signalling does not physiologically include an increase of the free intracellular zinc level.

### 2.5. Simultaneous Stimulation Promotes Activation and Internalisation of the EGFR

**Figure 5:**
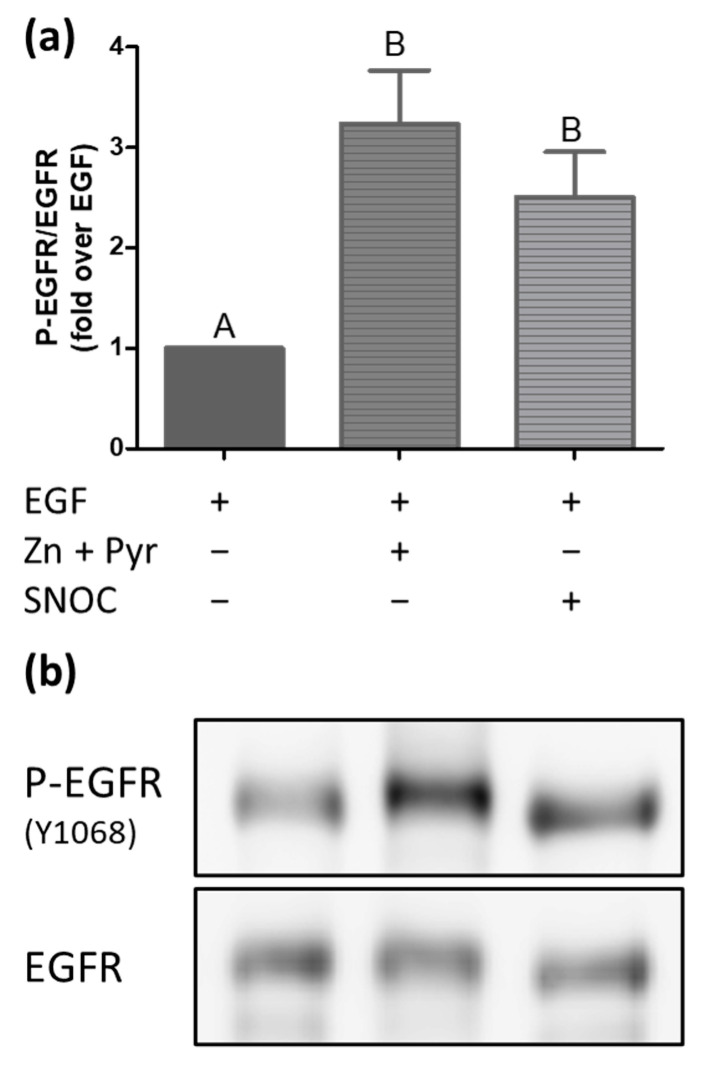
Zinc enhances EGF-induced EGFR phosphorylation. The effect of a simultaneous stimulation with zinc (Zn), pyrithione (Pyr) and EGF or SNOC and EGF on the tyrosine 1068 phosphorylation of the EGFR is shown. A549 cells were stimulated for 60 min with zinc (50 µM), pyrithione (0.5 µM) and EGF (25 ng/mL) or SNOC (2 mM) and EGF (25 ng/mL). Phosphorylation was quantified by Western blotting. Data of *n* = 12 independent experiments are shown as means + SEM (**a**). One representative Western blot is presented (**b**). Statistical significances were calculated with one-way Anova and Tukey’s post-hoc test (*p* < 0.05). Means not sharing any letter are significantly different (A, B).

**Figure 6:**
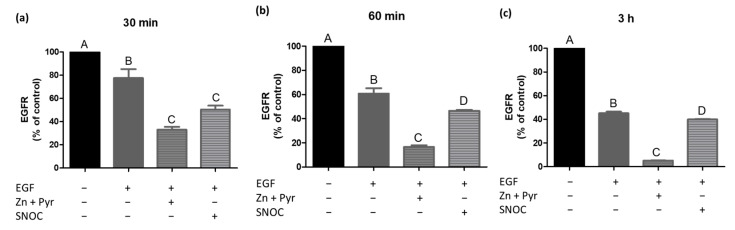
Zinc supports EGF-induced reduction of EGFR surface expression. The effect of a simultaneous stimulation with zinc (Zn, 50 µM), pyrithione (Pyr, 0.5 µM) and EGF (10 ng/mL) or SNOC (2 mM) and EGF (10 ng/mL) on the surface expression of the EGFR is shown. A549 cells were stimulated for 30 min (**a**), 60 min (**b**) or 3 h (**c**) and measurements were performed by using flow cytometry. Data of *n* = 3 independent experiments are shown as means + SEM. Statistical significances were calculated with one-way Anova and Tukey’s post-hoc test (*p* < 0.05). Means not sharing any letter are significantly different (A–D).

As the EGF-induced EGFR activation is not connected to an intracellular zinc increase, effects are probably explained by different underlying mechanisms. Possible consequential additive effects were thus examined by combined stimulations as a subsequent step (Figure 5 and Figure 6).

The effects of stimulation with zinc and pyrithione or SNOC in combination with EGF on EGFR phosphorylation are presented in Figure 5a. The receptor phosphorylation induced by simultaneous stimulation with EGF, zinc and pyrithione was three times as high as the activation induced by EGF alone. Although less pronounced, concurrent treatment with EGF and SNOC also led to a further increased phosphorylation of the EGFR, which was more than twice as high as effects observed for single treatments.

Figure 6a–c shows the receptor endocytosis after 30 min (a), 60 min (b) and 3 h (c) of simultaneous stimulation with zinc, pyrithione and EGF or SNOC in combination with EGF. At each time point the combined stimulations had a significantly stronger internalisation effect, compared to the single incubation with EGF. Equal to Figure 3a–c, endocytosis slowed down over the time. SNOC in combination with EGF caused a total receptor endocytosis of 60%. Although significant, the effects of SNOC were not as impressive as when zinc and pyrithione were added to the A549 cells in combination with EGF, which led to an almost complete internalisation of the EGFR (95%) within 3 h.

In summary, simultaneous stimulations significantly promote activation as well as internalisation of the EGFR in A549 cells.

## 3. Discussion

Former work suggested that a high intracellular free zinc level mediates a phosphorylation of the EGFR with subsequent activation of the MAPK cascade and the PI3K/Akt signalling [10,11,19]. So far published reports mainly concentrated on the impact of zinc on the signalling cascades downstream of the EGFR and further proposed that the mechanisms of zinc-initiated EGFR transactivation vary considerably between different cell types. The presented study confirms the zinc-mediated receptor transactivation for pulmonal cells and is, in contrast to previous works, the first focusing on the effects of zinc on the EGFR endocytosis. By comparing results obtained for two different sources of zinc and for single as well as for combined stimulations in the same cellular system, zinc’s effects on EGFR activation and internalisation were systematically addressed in this study.

In contrast to the examinations in human epidermoid cells [7] and in mouse fibroblast cells [6], studies in primary human bronchial epithelial cells suggest the involvement of the EGFR tyrosine kinase in the transactivating process by zinc [8,9]. The inhibition of tyrosine phosphatases by zinc [9] or a zinc-mediated cleavage of membrane bound EGFR ligands [8] provoke an activation of the receptor tyrosine kinase with subsequent receptor autophosphorylation. As opposed to our work, in the studies mentioned above zinc concentrations of up to 500 µM were added without ionophore, solely extracellularly and without checking changes of the intracellular zinc levels for varying stimulation periods, ranging from 5 min to 4 h [6,7,8,9]. By examining tyrosine 1068 as a main autophosphorylation site of the EGFR, the here presented study confirms the activating effect of zinc on this receptor for the human airway epithelial cell line A549, which was as strong as the phosphorylation effect of EGF itself (Figure 2). As the phosphorylation effects are consistent with the findings for primary human airway epithelium cells [8,9], these results further emphasize the suitability of A549 cells for EGFR investigations. The free intracellular zinc level is documented to be in a pico- to low nanomolar range and small fluctuations are sufficient to alter intracellular signalling [20,21]. Taylor et al. found that an increased expression of ZIP7 in the endoplasmic reticulum was related to an enhanced intracellular zinc release in breast cancer cells, which augmented activation of the EGFR and could explain aggravated anti-tumour-therapy [22]. In addition to zinc release through ZIP7, nitric oxide (NO), superoxide and peroxide are involved in the oxidation of zinc binding proteins, transmitting redox signals into zinc signals [14,23,24]. A second possibility to elevate the free intracellular zinc concentration is thus the induction of an intracellular zinc release via the NO donor SNOC, which also led to a significant activation of the EGFR (Figure 2). The increase in EGFR phosphorylation mediated by SNOC-induced zinc release remained lower compared to the phosphorylation effect of zinc and pyrithione, which could be well explained by the difference of intracellular zinc levels achieved in both settings (Figure 1). Thus, independent of the zinc source, an elevation of the free intracellular zinc concentration led to a dose-dependent activation of the EGFR in A549 cells, probably by the inhibition of phosphatases, as described [20] and supports the results from previous studies, which had uncovered the effect of zinc on down-stream processes induced by EGFR ligation. Moreover, as SNOC exclusively mobilise zinc from intracellular zinc sources, an EGFR activation from the extracellular side is highly unlikely. This also largely rules out the possible receptor transactivating effect of the G-protein coupled receptor ZnR/GPR39. The ZnR/GPR39 is expressed by various tissues, activated by extracellular zinc ions [25] and has shown to be capable to transactivate the EGFR [26].

Studies examined a direct effect of NO on the EGFR, as S-nitrosylation of amino acids could also be linked to changes of the receptor activity. While numerous investigations have proven an inhibitory effect of NO donors on the EGFR and its signalling [27,28,29,30], a comparably vast body of evidence revealed exactly the opposite [31,32,33]. Since the molecular mechanisms of the activating and inactivating effects of NO are still in the focus of discussions, we grant at this point, that a direct, zinc-independent effect of NO on the EGFR and on enzymes being involved in the EGFR activation cannot be fully excluded. Lee et al. observed an activation of the EGFR in A549 cells in response to the NO source S-nitroso-N-acetylpenicillamine (SNAP). They hypothesized a direct activation of the EGFR by NO but also admitted that other cellular compounds may be involved and act as messenger [33]. While we showed a significant receptor activation after 60 min of SNOC stimulation, the strongest receptor phosphorylation in response to SNAP was obtained after 15 min [33]. This deviation could be explained by varying zinc concentrations received using different NO donors and amounts. Even though experimental settings were different, regarding the potential of zinc to transactivate the EGFR, together with the presented zinc release as a direct result of NO exposure in the same cell line (Figure 1), a role of zinc as second messenger in this context is likely.

Although the zinc-mediated EGFR transactivation was suggested before, the degree of zinc-induced receptor endocytosis was surprisingly never a focus of previous work. Endocytosis controls amongst others duration and strength of signalling events and is thus an important mechanism to manage an appropriated cell response [3]. Indeed, all typical ligands of the EGFR vary a lot in their speed and extent of initiated receptor internalisation [34] and beyond that, previous works have already discovered new activators, inducing EGFR phosphorylation even without receptor internalisation [35,36]. For example, an EGFR activation triggered by H_2_O_2_ in A549 cells has shown to be uncoupled from receptor endocytosis, as phosphorylated receptors remained on the cell surface [37]. The latter suggests EGFR activation and internalisation to be independent at times, which emphasizes the importance to highlight zinc’s effects on both processes, separately.

While the receptor phosphorylation was detectable after 60 min of stimulation (Figure 2), receptor internalisation already showed considerable dimensions after 30 min (Figure 3a). This confirms receptor signalling and endocytosis to be parallel processes, as previously described [38,39]. Furthermore, an earlier work of our own laboratory excluded a direct influence of zinc on the membrane fluidity in A549 cells, which underlines the EGFR activation by phosphorylation as most important mechanism triggering the receptor internalisation [40]. The continuous stimulation of A549 cells with zinc and pyrithione for up to 3 h led to a time-dependent quick endocytosis of the EGFR and showed a greater extent than the EGF exposure itself, suggesting a big intervening potential of a high intracellular zinc concentration on the internalisation process (Figure 3a–c). Surprisingly, even the mild zinc increase induced by SNOC caused a pronounced EGFR internalisation, in particular after a SNOC exposure of 30 min (Figure 3a). Since a recent work found that intracellular zinc concentrations tend to be lower when measured with a flow cytometer instead of a well plate reader, the actual free intracellular zinc concentrations are probably lower than determined in our experiments [41]. The combination of zinc and pyrithione incubation is frequently used to get an impression of the maximal potential of zinc, although it is known that long-term incubation with zinc together with its ionophore can result in intoxication of the cells. By contrast, experiments performed with SNOC are much closer to the physiological conditions and results should thus be taken into account for in vivo considerations.

With all stimulants tested here the internalisation extent slowed down over the time. This might be caused by a repeated endocytosis of already recycled receptors, as a parallel begin of EGFR recycling was previously observed after 30 min stimulation [34]. In addition, the chosen EGF concentration (10 ng/mL) was suggested to allow at least a partial receptor recycling [42]. Thus, a receptor recycling could be conceivable to occur in the case of zinc as well. Regarding the SNOC-induced internalisation, a further explanation should be mentioned. Monitoring the SNOC-induced changes in intracellular free zinc concentrations, a normalization towards the basal zinc level started 30 min after SNOC had been added (Figure 1). This might be caused by the short half-life and thus linked consumption of SNOC [16]. In regard to the kinetic of the intracellular zinc release and the thereto relating presumed decrease of EGFR transactivation, the slowed receptor internalisation can be explained by this as well.

Thus far, results described here showed a close similarity of changes induced by zinc compared to the EGF effects, raising the impression that a zinc wave, induced by EGF, might be physiologically involved in the intracellular signalling of the EGFR. Previous works in different cell types showed contradicting results, partly confirming [18,43] as well as partly rejecting this hypothesis [44]. We also excluded the involvement of a zinc wave in the EGF-induced EGFR signalling for A549 cells (Figure 4), which suggests the intracellular zinc homeostasis to be an independent, synergistic modulator of EGFR-initiated growth signals. Our results support the findings from other studies, that zinc is a major player during regulation of EGFR-induced signalling. There is a complex signalling network involving transactivation of the EGFR by G protein-coupled receptors, cytokine receptors, oestrogen receptors, integrins, or stress-inducing agents [45]. As ligation of some of those receptors indices alterations in intracellular zinc levels, zinc might be a major player during the transactivation of the EGFR by other stimulants as well [46,47]. This should be addressed in future studies.

As the continuous expression of high levels of EGFR on the surface of pulmonal cells is associated with lung cancer and pulmonal fibrosis, the finding that zinc does not only influence receptor phosphorylation and downstream signalling, but also receptor endocytosis may be important for future drug development for those diseases [4,5].

In accordance with the findings of afore described experiment, the combined stimulation with zinc and EGF (Figure 5 and Figure 6) showed a significantly enhanced receptor phosphorylation and internalisation, compared to the sole stimulation with EGF. These results underline the physiological role of zinc to promote naturally triggered EGFR activation and endocytosis, already with small intracellular zinc increases. As mentioned above, this finding is of high relevance since the EGFR is a key player in the complex signalling network of various classes of cell-surface receptors. Similar to the results of the endocytosis assays presented in Figure 3, the combined incubation with SNOC and EGF caused initially a strong receptor endocytosis (Figure 6a), but subsequently adapted to the internalisation extent of the EGF incubation alone (Figure 6c). This is again consistent with the SNOC-induced zinc wave in the cell, suggesting a decreasing influence of zinc on the receptor internalisation after 30 min (Figure 1b). These observations point to a fine-tuning effect of zinc on this receptor and indicate that zinc might play a role during transactivation of the EGFR by other stimuli.

## 4. Materials and Methods

### 4.1. Cell Culture and Cell Stimulation

Human lung alveolar carcinoma cell line A549 was cultured in RPMI-1640 (Sigma-Aldrich, Steinheim, Germany), supplemented with 10% heat inactivated FCS (Lonza, Verviers, Belgium), 2 mM L-glutamine, 100 U/mL potassium penicillin and 100 µg/mL streptomycin sulfate (all from Sigma-Aldrich). For every assay, cells growing to 90% confluence, were starved for 24 h in RPMI-1640 containing 1% heat inactivated FCS, 2 mM L-glutamine, 100 U/mL potassium penicillin and 100 µg/mL streptomycin sulfate in culture flasks or 96-well plates, before starting examination. Subsequently, cells were stimulated with RPMI-1640 (medium), zinc sulfate, sodium pyrithione (both from Sigma-Aldrich), S-nitrosocysteine (SNOC), recombinant human EGF (ImmunoTools, Friesoythe, Germany), ionomycin (Sigma-Aldrich) and combinations of the named stimulants, as indicated in the figure legends. To generate SNOC, L-cysteine and NaNO_2_ (both from Sigma-Aldrich) were mixed instantly before usage [16]. Induction of apoptosis by any of the stimulants was excluded by trypan blue and annexin staining.

### 4.2. Measurement of the Free Intracellular Zinc Concentration

Free intracellular zinc levels were measured by using the cell permeable and zinc selective probe FluoZin-3 AM (Invitrogen, Darmstadt, Germany), as previously described [48]. The zinc-dependent fluorescence changes were recorded either in a kinetic measurement, starting immediately after adding the stimulants, by using a Spark multimode reader (temperature: 37 °C, excitation: 485 nm, emission: 535 nm) (Tecan, Crailsheim, Germany) or measured as end point determinations by using flow cytometry (FACSCalibur, BD Bioscience, Heidelberg, Germany). The intracellular zinc concentration was determined by the following formula: $\left[Zn^{2+}\right]=K_{D}\times \frac{F-F_{\min}}{F_{\max}-F}$, using a dissociation constant (K_D_) of 8.9 nM for the Zn/FluoZin-3 AM complex. F_max_ and F_min_ values were reached by incubating cells with 100 µM zinc sulfate in combination with 5 µM of its ionophore sodium pyrithione (F_max_) or with 100 µM of its chelator *N*,*N*,*N*,*N*-tetrakis(2-pyridylmethyl)-ethylenediamine (TPEN) (Sigma-Aldrich) (F_min_), respectively.

### 4.3. Sample Preparation and Western Blotting

After the cell stimulation, tubes were centrifuged, supernatants were discarded and cell pellets were lysed in sample buffer (65 mM Tris-HCl (Sigma-Aldrich), 25% glycerol (Fisher Scientific GmbH, Schwerte, Germany), 2% SDS, 0.01% bromophenol blue, 1% β-mercaptoethanol, 1 mM sodium orthovanadate (all from Sigma-Aldrich)). Samples were sonicated for 30 s and heated up for 5 min at 95 °C.

Apart from each sample, at least one coloured standard (New England BioLabs, Frankfurt am Main, Germany) was applied in the pockets of an 8.5% polyacrylamide gel. Following the protein separation, proteins were transferred to a nitrocellulose membrane (Bio-Rad, Munich, Germany). Subsequently, membranes were blocked with TBS-T (20 mM Tris-HCl, 136 mM NaCl (AppliChem, Darmstadt, Germany), 0.1% Tween 20 (Sigma-Aldrich)) containing 5% powdered milk (Saliter, Obergünzburg, Germany) for 1 h. Primary antibodies against the total EGFR protein and the phosphorylated tyrosine 1068 (both from Cell Signaling Technology, Frankfurt am Main, Germany) were diluted 1:1000 in TBS-T containing 5% bovine serum albumin (BSA, AppliChem). Membranes were incubated over night at 4 °C with the primary antibodies. The next day membranes were washed with TBS-T, before incubating in secondary antibody. Secondary antibody, a HRP-coupled anti-rabbit IgG (Cell Signaling Technology), was diluted 1:2000 in TBS-T containing 5% powdered milk and membranes were incubated for at least 3 h at room temperature. To visualize the protein bands, LumiGlo reagent (Cell Signaling Technology) was used for the detection of the total EGFR protein and ECL^TM^ Prime Western Blotting Detection Reagent (Sigma-Aldrich) was used for the detection of the phosphorylated tyrosine 1068 of the EGFR. Luminescence was analysed by using a LAS 3000 (Fujifilm Lifescience, Düsseldorf, Germany) and quantified with ImageJ (National Institutes of Health, Bethesda, MD, USA).

### 4.4. Measurement of EGFR Surface Expression

Following the stimulation, cells were incubated with Alexa Flour 647 coupled anti-human EGFR antibody or Alexa Fluor 647 anti-mouse IgG1 κ (both from BioLegend, San Diego, CA, USA), as isotype control, for 15 min in the dark at room temperature. Subsequently, cells were washed, resuspended in phosphate-buffered saline (PBS) (Sigma-Aldrich) and the amount of receptor on the cell surface was quantified by using flow cytometry. Data analysis was done by using Cellquest software 3.0 (BD Bioscience).

### 4.5. Statistics

Besides a paired Student’s *t*-test for single comparisons, one-way ANOVA combined with Tukey’s post-hoc test was used for multiple comparisons, both calculated with Graph Pad Prism software (version 5.01, Graph Pad software, San Diego, CA, USA). For both tests *p*-values below 0.05 were indicated to be statistically significant. Significances are illustrated in the figures by * (*p* < 0.05), ** (*p* < 0.01) or *** (*p* < 0,001), for a paired Student’s *t*-test (Figure 1). To indicate all significant differences between the different data sets as calculated by one-way ANOVA (*p* < 0.05), we chose the letters A–D (for a detailed explanation, see [49]). Significantly different datasets do not share the same letters (Figure 2, Figure 3, Figure 4, Figure 5 and Figure 6).

## 5. Conclusions

The results of the here presented work affirm the suggested zinc-mediated EGFR transactivation for A549 cells and further provide first knowledge of zinc’s influence on receptor internalisation. An extracellular zinc addition as well as an intracellular zinc release showed similar effects, revealing a dose dependent phosphorylation and a quick and strong, dose- and time-dependent internalisation of the EGFR. Even small zinc fluctuations caused receptor endocytosis, which awards zinc to have a physiological, fine-tuning and controlling effect on the EGFR. Short term strengthening of the receptor signalling promotes cell growing signals and could thus be helpful in the regulation of lung epithelial regeneration and wound healing. The fate of the internalised receptors determines the actual resulting pro-mitogenic potential of zinc. Thus, to elucidate zinc’s effects fully and conclusively in relation to the EGFR, future works should in particular concentrate on receptor endocytosis pathways and on the verification of the results in vivo.

## Data Availability

The data presented in this study are available on request from the corresponding author.

## Abbreviations

EGF	Epidermal growth factor
EGFR	Epidermal growth factor receptor
SNOC	S-nitrosocysteine
SNAP	S-nitroso-*N*-acetylpenicillamine
NO	Nitric oxide
Zn	Zinc
Pyr	Pyrithione
TPEN	*N*,*N*,*N*,*N*-tetrakis(2-pyridylmethyl)-ethylenediamine
BSA	Bovine serum albumin
PBS	Phosphate-buffered saline
